# Comparison of Physical Frailty Assessments in Heart Failure With Preserved Ejection Fraction

**DOI:** 10.1016/j.jacadv.2024.101395

**Published:** 2024-12-26

**Authors:** Omar Zainul, Dylan Marshall, Jennifer D. Lau, Brooke Kelly, Kate Zarzuela, Abdulla Damluji, Ambarish Pandey, Amy M. Pastva, Parag Goyal

**Affiliations:** a Department of Medicine, Weill Cornell Medicine, New York, New York, USA; b Program for the Care and Study of the Aging Heart, Weill Cornell Medicine, New York, New York, USA; c Division of Cardiology, Johns Hopkins University School of Medicine, Baltimore, Maryland, USA; d Inova Center of Outcomes Research, Inova Heart and Vascular Institute, Falls Church, Virginia, USA; e Division of Cardiology, Department of Internal Medicine, University of Texas Southwestern Medical Center, Dallas, Texas, USA; f Departments of Orthopedic Surgery (Physical Therapy Division), Medicine, and Population Health Sciences, Duke University School of Medicine, Durham, North Carolina, USA; g Claude D. Pepper Older Americans Independence Center, Duke University School of Medicine, Durham, North Carolina, USA

**Keywords:** frailty, heart failure with preserved ejection fraction, morbidity, mortality, prognosis

## Abstract

**Background:**

Frailty is a known determinant of poor clinical outcomes in heart failure with preserved ejection fraction (HFpEF). However, prevalence estimates and effect sizes vary in part due to multiple tools available to measure frailty.

**Objectives:**

This study aimed to compare the prevalence and prognostic value of six commonly used frailty assessments in adults with HFpEF.

**Methods:**

We examined 203 outpatients with HFpEF seen at Weill Cornell Medicine from June 2018 to August 2022. The following frailty scales were compared: the Clinical Frailty Scale (CFS), the Fatigue, Resistance, Ambulation, Illnesses, and Loss of Weight scale (FRAIL) scale, the 5-m gait speed test, the 5 timed sit-to-stand test, hypoalbuminemia, and the modified body mass index score. The primary endpoint was a 1-year composite of all-cause mortality and hospitalization. Cox proportional hazard models were used to examine the association between frailty and the primary endpoint, adjusting for race and the MAGGIC (Meta-Analysis Global Group in Chronic) heart failure prognostic risk score.

**Results:**

The median age was 76.7 years (IQR: 69.7-83.9 years). The prevalence of frailty ranged from 21.2% (hypoalbuminemia) to 77.8% (5 timed sit-to-stand) and increased with advancing HFpEF severity. Of the 6 frailty assessments, the CFS (HR: 2.83; 95% CI: 1.61-4.98, *P* < 0.001), FRAIL scale (HR: 1.96; 95% CI: 1.25-3.07, *P* = 0.004), and 5-m gait speed test (HR: 2.80; 95% CI: 1.50-5.25, *P* = 0.001) were associated with adverse outcomes in the multivariate analysis.

**Conclusions:**

Frailty assessments yield a wide range of prevalence estimates and vary in their associations with clinical outcomes. The CFS, FRAIL scale, and the 5-m gait speed tests demonstrated associations with adverse outcomes and may thus be reasonable tools for routine use in patients with HFpEF.

Frailty has emerged as an important phenomenon in heart failure with preserved ejection fraction (HFpEF) for multiple reasons. First, frailty is highly prevalent in HFpEF: the prevalence of frailty in individuals with HFpEF ranges from 50% to 94%.^1^^,^^2^ Second, and relatedly, the pathophysiology of frailty and HFpEF are closely intertwined^2^— both frailty and HFpEF are driven by a chronic inflammatory process associated with oxidative stress and mitochondrial dysfunction which causes metabolic dysfunction, cellular senescence, and cellular necrosis.^2^^,^^3^ Third, frailty is a known risk factor for adverse events in HFpEF,^4^, ^5^, ^6^ conferring up to a 2-fold increased risk for death.^5^^,^^7^ Given these known facts, frailty assessments are now recommended by international heart failure guidelines in the care of patients with HFpEF.^8^, ^9^, ^10^

While the clinical relevance of frailty in HFpEF is no longer debated, there is a lack of consensus on how best to assess frailty. This is especially problematic since over 60 unique frailty evaluation tools exist.^11^ As different clinicians use different frailty assessments, the reported prevalence of frailty among patients with HFpEF varies over a wide range.^12^ It is also unknown which tool offers the greatest prognostic value. To date, there have been limited head-to-head comparison studies on the prognostic value of multiple frailty assessments in heart failure,^13^^,^^14^ and none to our knowledge specifically in HFpEF. As such, the objective of this study was to compare the prevalence and prognostic value of six commonly used frailty assessments among HFpEF populations in the clinical setting.

## Methods

### Study sample

This study was conducted at a dedicated outpatient HFpEF Program at Weill Cornell Medicine from June 2018 through August 2022. The patients cared for at this program include those with a prior diagnosis of HFpEF and those seeking evaluation for a potential new diagnosis of HFpEF. As part of routine care in this program, patients undergo multidomain geriatric assessments^15^^,^^16^ guided by a recent expert recommendation.^17^ These assessments include routine evaluations of frailty among other geriatric conditions. These data are inputted into a registry, which has been approved by a local ethics committee.

For the current study, we examined patients with a clinical diagnosis of HFpEF. Consistent with guidelines, HFpEF was defined as: 1) presence of HF symptoms based on Framingham criteria^18^; 2) preserved left ventricular ejection fraction (LVEF) ≥50%^19^; and 3) absence of alternative HF etiologies, including severe valvular disease, hypertrophic cardiomyopathy, pericardial disease, pulmonary arterial hypertension, cardiac amyloidosis, or other infiltrative diseases.

### Primary factor of interest

We examined six frailty scales routinely administered during the first visit (baseline) to the WCM HFpEF Program (Central Illustration), including the Rockwood Clinical Frailty Scale (CFS), the Fatigue, Resistance, Ambulation, Illnesses, and Loss of Weight scale (FRAIL scale), the 5-meter gait speed test, the 5 timed sit-to-stand test (5-STS), serum albumin values, and the modified body mass index (mBMI) score. The CFS is scored from 1 to 9 based on a semiquantitative evaluation of the patient’s symptoms, mobility, inactivity, exhaustion, disability for basic activities of daily living (ADL), and instrumental ADL's. Frailty was classified as a CFS score ≥4 as the prognostic impact of this cutoff has been validated in a hospitalized HFpEF cohort.^20^ The FRAIL scale is a multidomain frailty assessment developed and validated by Morley et al,^21^ and has demonstrated prognostic value in multiple HF cohorts.^13^^,^^22^ The FRAIL scale incorporates the following domains: fatigue, resistance, ambulation, number of comorbidities, and weight loss. One point is given for each component: a score of 3 to 5 represents frail; 1 to 2 represents pre-frail; and a score of zero represents robust (or nonfrail).^21^ Patients with scores ≥3 on the FRAIL scale were deemed frail in this study. For the 5-m gait speed test, patients were timed as they walked 5 meters at usual speed beginning from a standing stationary position. This sequence was repeated 3 times, with rest between each attempt; the average of the three 5-m gait speeds was recorded. Patients with gait speeds ≥6 seconds were classified as frail in this study as this cutoff has been validated as an independent predictor of morbidity and mortality among patients with cardiovascular diseases.^23^^,^^24^ The 5-STS test is an assessment of functional lower extremity strength. Patients were timed as they stood up and sit down five times with their arms folded starting from a seated position; times ≥15 seconds was used as the cutoff for frailty. The 5-STS test has independently been shown to be a predictor of falls and a decline in ADL in elderly patients.^25^^,^^26^ Of note, the 5-STS on its own has not been studied in the context of frailty or as a prognostic indicator in HF. Hypoalbuminemia, defined as serum albumin values <3.5 g/dL, has been described as a marker of frailty^27^ and has demonstrated conflicting results for predicting outcomes in HFpEF cohorts.^28^, ^29^, ^30^ Serum albumin values were obtained at patients’ baseline visit; albumin <3.5 g/dL was used as the cutoff for frailty. The mBMI is a newly described frailty index that represents the product of BMI and serum albumin level and has been associated with adverse outcomes in several cardiovascular disease states.^31^^,^^32^ Since there are no current accepted cutoff values for frailty, patients with mBMI scores under 50% of the median were considered frail in this study.

**Central Illustration:**
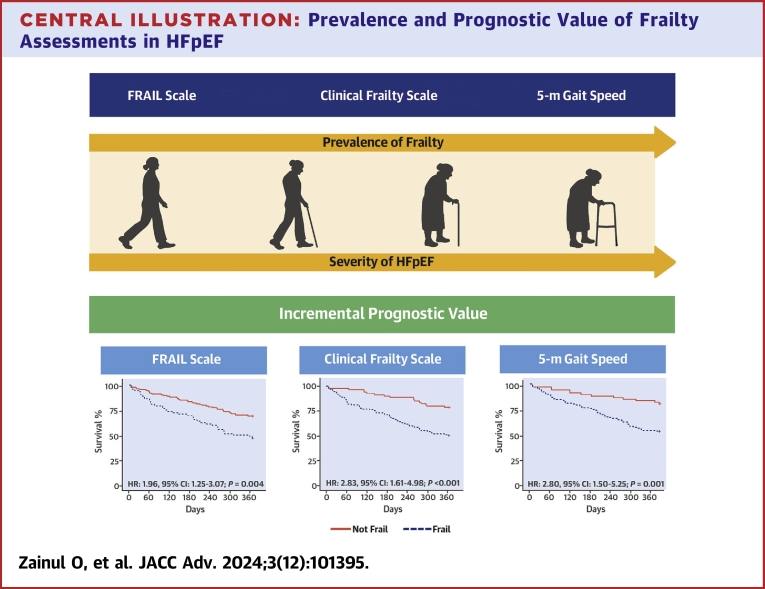
**Prevalence and Prognostic Value of Frailty Assessments in HFpEF** Kaplan-Meier curves for the composite outcome of all-cause mortality and all-cause hospitalization in patients with (Clinical Frailty Scale ≥4, FRAIL score ≥3, 5-m gait speed ≥6 seconds) and without (Clinical Frailty Scale <4, FRAIL score <3, 5-m gait speed <6 seconds) frailty. HFpEF = Heart Failure With Preserved Ejection Fraction; other abbreviation as in Figure 1.

### Primary outcome

The primary outcome was a composite of all-cause mortality and all-cause nonelective hospitalization over a 1-year follow-up period after baseline administration of the frailty assessments. Outcomes were ascertained based on a review of the electronic medical record. The electronic medical record used at Weill Cornell Medicine incorporates nearly all encounters within the health care system and provides extensive access to health care encounters outside the institution. Moreover, since most patients included in this study were seen multiple times at the Weill Cornell Medicine HFpEF Program throughout this study, there were ample encounters within the electronic medical record to ascertain outcomes.

### Statistical analysis

Continuous variables were expressed as median (IQR): the differences were compared using the Wilcoxon rank sum test. Categorical variables were expressed as count (percentage) and assessed using Pearson’s chi-squared or Fisher’s exact test. Frailty scales were analyzed in their dichotomous form based on their respective cutoffs. The prevalence of frailty was stratified by the median Meta-Analysis Global Group in Chronic (MAGGIC) heart failure risk prognostic scores and NYHA functional classes. To examine concordance among the frailty tools, we calculated phi coefficients for each pair of frailty tools.

Kaplan-Meier curves with log-rank tests were used to examine crude associations in outcomes among those with and without frailty. To evaluate associations of frailty with the composite outcome, we conducted Cox proportional hazards model analysis adjusting for demographics and severity of illness. To adjust for these important confounders, we incorporated the MAGGIC heart failure prognostic risk score. The MAGGIC prognostic risk score has been validated in HFpEF as a predictor of morbidity and mortality,^33^ and includes the following variables: age, sex, diabetes mellitus, chronic obstructive pulmonary disease, smoking status, BMI, systolic blood pressure, creatinine, LVEF, first HF diagnosis within prior 18 months of baseline visit, NYHA functional class, and current beta-blocker, angiotensin-converting enzyme inhibitor, or angiotensin receptor blocker usage. Since race is not included in the MAGGIC score and may be an important confounder, we adjusted for White vs non-White race. Since multiple frailty tools were examined (6), we included a Bonferonni adjustment into our models—a *P* value of <0.0083 was considered statistically significant. Logistic regression analysis was used to examine the linear relationship of frailty assessment scores with the log-predicted odds for adverse outcomes. The proportional hazards assumption was checked using Schoenfeld residuals against time. Statistical analyses were performed using R, 4.3.1 (R Foundation for Statistical Computing, Vienna, Austria).

## Results

A total of 203 patients were included in this study, with a median follow-up time of 1 year; 17 patients were lost to follow-up (no available data to indicate vital status or interim hospitalization events) and were not included in further analyses. The median age was 76.7 years (IQR: 69.7-83.9 years), and the majority were female (66.5%) and White (66.0%). The median LVEF was 63.0% (IQR: 59.0% to 68.0%). The most common comorbid conditions were hypertension (80.3%), obesity (53.2%), atrial fibrillation (47.3%), and diabetes mellitus (39.9%). Most patients had either NYHA functional class II (39.4%) or NYHA functional class III (52.7%) heart failure symptoms and the median MAGGIC score was 24.0 (IQR: 19.0-28.0). A total of 79 (38.9%) patients experienced the composite outcome of all-cause mortality and all-cause nonelective hospitalization over the 1-year follow-up. Patients who experienced the composite outcome had slightly higher baseline creatinine values than those who did not. Additionally, patients who experienced adverse outcomes had higher scores on the CFS, FRAIL scale, 5-m gait speed, and the 5-STS test and had lower serum albumin levels (Table 1).

**Table 1:** Baseline Study Population Characteristics Stratified by the 1-Year Event Occurrence for the Composite Outcome of All-Cause Mortality and All-Cause Hospitalization

	Overall (N = 203)	1-Year Adverse Event Occurrence		
		No (n = 124)	Yes (n = 79)	*P* Value^a^
Sociodemographics				
Age, y	76.7 (69.7-83.9)	76.1 (69.4-82.6)	77.8 (70.7-86.5)	0.23
Female	135 (66.5%)	84 (67.7%)	51 (64.6%)	0.64
Primary race				0.30
Caucasian	134 (66%)	85 (69%)	49 (62%)	
African American	33 (16%)	21 (17%)	12 (15%)	
Asian	9 (4.4%)	5 (4.0%)	4 (5.1%)	
Other	25 (12%)	11 (8.9%)	14 (18%)	
Unknown	2 (1.0%)	2 (1.6%)	0 (0.0%)	
Hispanic ethnicity	22 (10.8%)	11 (8.9%)	11 (13.9%)	0.26
Comorbid conditions				
Coronary artery disease	65 (32.0%)	38 (30.6%)	27 (34.2%)	0.6
Atrial fibrillation	96 (47.3%)	53 (42.7%)	43 (54.4%)	0.1
Diabetes mellitus	81 (39.9%)	47 (37.9%)	34 (43.0%)	0.47
Hypertension	163 (80.3%)	97 (78.2%)	66 (83.5%)	0.35
COPD	44 (21.7%)	24 (19.4%)	20 (25.3%)	0.31
Cancer	58 (28.6%)	37 (29.8%)	21 (26.6%)	0.62
Current smoker	3 (1.5%)	1 (0.8%)	2 (2.5%)	0.56
Overweight	55 (27.1%)	36 (29.0%)	19 (24.1%)	0.44
Obesity	108 (53.2%)	64 (51.6%)	44 (55.7%)	0.57
BMI (kg/m^2^)	30.5 (26.2-36.9)	30.3 (26.1-36.1)	30.9 (27.2-38.0)	0.24
Systolic BP (mm Hg)	131.0 (121.0-145.5)	131.5 (122.0-146.5)	130.0 (118.0-145.0)	0.56
Creatinine (mg/dL)	1.1 (0.8-1.4)	1.0 (0.8-1.3)	1.2 (0.9-1.6)	**0.02**
HF characteristics				
HF diagnosis in the past 18 mo	132 (65.0%)	82 (66.1%)	50 (63.3%)	0.68
NYHA functional class				0.24
I	11 (5.4%)	9 (7.3%)	2 (2.5%)	
II	80 (39.4%)	52 (41.9%)	28 (35.4%)	
III	107 (52.7%)	61 (49.2%)	46 (58.2%)	
IV	5 (2.5%)	2 (1.6%)	3 (3.8%)	
LVEF (%)	63.0 (59.0-68.0)	62.0 (59.0-67.3)	64.0 (59.5-69.5)	0.55
MAGGIC score	24.0 (19.0-28.0)	23.0 (18.0-27.0)	25.0 (20.5-29.0)	0.08
Baseline medication usage				
Beta-blockers	120 (59.1%)	67 (54.0%)	53 (67.1%)	0.06
ACEI/ARB	84 (41.4%)	50 (40.3%)	34 (43.0%)	0.70
Statins	152 (74.9%)	96 (77.4%)	56 (70.9%)	0.30
Digoxin	10 (4.9%)	6 (4.8%)	4 (5.1%)	>0.99
Anticoagulants	92 (45.3%)	50 (40.3%)	42 (53.2%)	0.07
Antiplatelet medications	14 (6.9%)	10 (8.1%)	4 (5.1%)	0.41
Frailty assessment scoring				
Clinical Frailty Scale	4.0 (3.0-5.0)	3.5 (3.0-4.0)	5.0 (4.0-6.0)	**<0.001**
FRAIL scale	2.0 (1.0-3.0)	2.0 (1.0-3.0)	2.0 (2.0-3.0)	**<0.001**
5-meter gait speed (s)	6.4 (5.3-8.1)	6.2 (5.3-7.5)	6.9 (5.8-9.1)	**0.015**
5-STS test (s)	16.8 (14.2-21.3)	16.6 (13.3-19.8)	19.4 (15.1-23.2)	**0.04**
Albumin level (g/dL)	3.8 (3.5-4.0)	3.9 (3.6-4.1)	3.8 (3.4-4.0)	**0.015**
Modified BMI score	116.4 (94.6-135.8)	117.4 (96.9-134.4)	115.6 (94.0-142.9)	0.98

**Bolded***P* Values denotes statistical significance. Values are median (IQR) or n (%).

ACEI = angiotensin-converting enzyme inhibitor; ARB = angiotensin receptor blocker; BMI = body mass index; BP = blood pressure; COPD = chronic obstructive pulmonary disease; dL = deciliter; FRAIL = Fatigue, Resistance, Ambulation, Illnesses, and Loss of Weight; HF = heart failure; LVEF = left ventricular ejection fraction; MAGGIC = Meta-Analysis Global Group in Chronic; 5-STS = 5 timed sit-to-stand.

a Wilcoxon rank sum test; Pearson’s chi-squared test; Fisher’s exact test.

**Figure 1:**
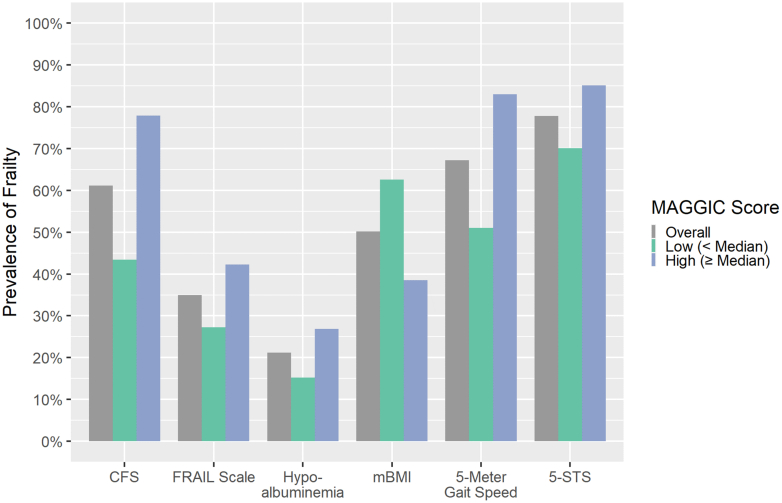
**Prevalence of Frailty Overall and Stratified by Median MAGGIC Scores** CFS = Clinical Frailty Scale; FRAIL = Fatigue, Resistance, Ambulation, Illnesses, and Loss of Weight; MAGGIC = Meta-Analysis Global Group in Chronic Heart Failure; mBMI = modified body mass index; 5-STS = 5 timed sit-to-stand.

**Figure 2:**
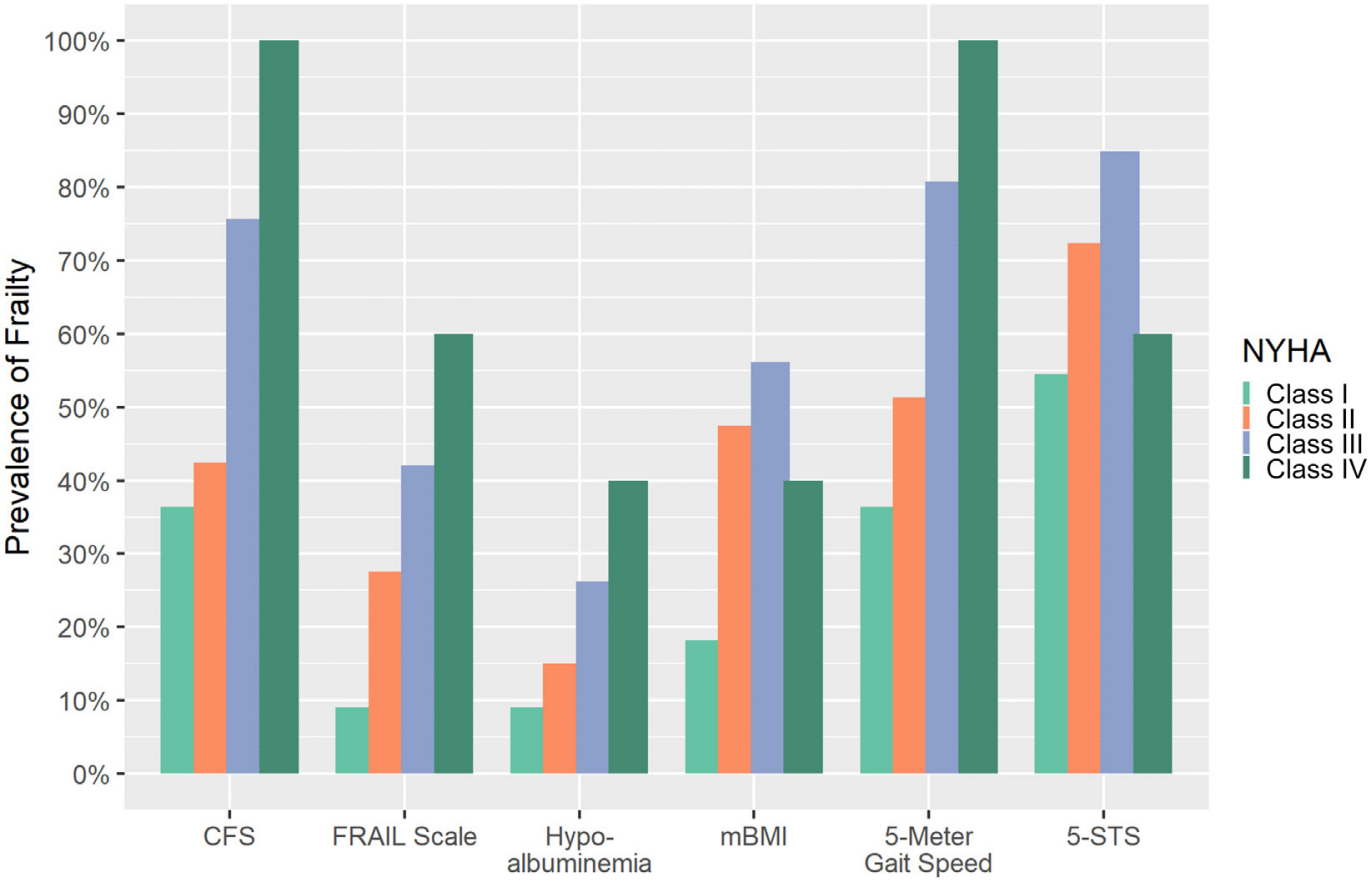
**Prevalence of Frailty Stratified by NYHA Functional Class** Abbreviations as in Figure 1.

**Figure 3:**
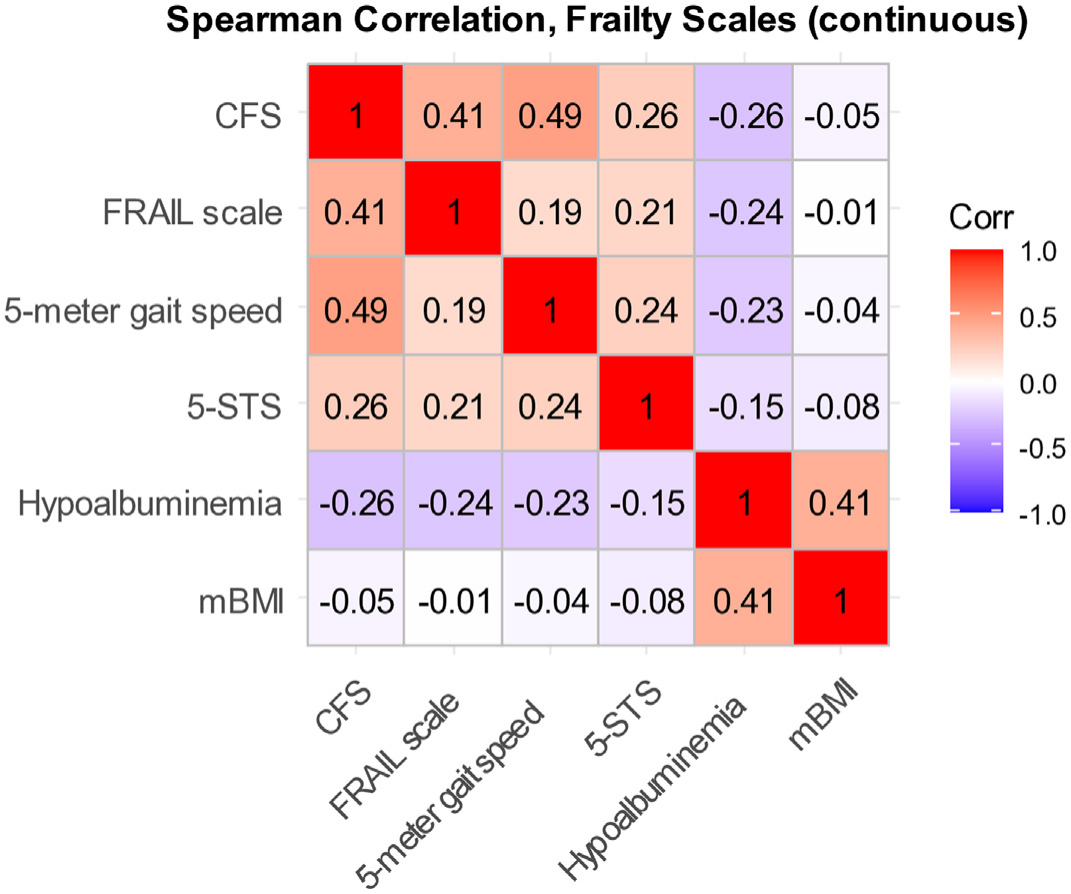
**Concordance Among Frailty Tools** Abbreviations as in Figure 1.

Among the 6 frailty assessments, the prevalence of frailty ranged from 21.2% (hypoalbuminemia) to 77.8% (5-STS) (Figure 1). The prevalence of frailty was higher among those with MAGGIC scores above the median for all frailty assessments (Figure 1). The prevalence of frailty in relation to NYHA functional class increased in a stepwise fashion with higher NYHA functional classes for all frailty assessments except for the 5-STS (Figure 2). The concordance among tools is shown in Figure 3; none had a concordance of >0.49.

**Figure 4:**
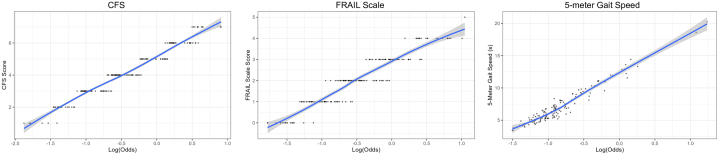
**Logistic Regression Analysis of Frailty Assessment Scores and the Log Predicted Odds for the 1-Year Composite Outcome of All-Cause Mortality and All-Cause Hospitalization** Abbreviations as in Figure 1.

In an unadjusted analysis, 4 of the 6 frailty assessments were associated with the composite outcome: the CFS (HR: 2.93; 95% CI: 1.71-5.02, *P* < 0.001), the FRAIL scale (HR: 2.07; 95% CI: 1.33-3.22, *P* = 0.001), the 5-m gait speed test (HR: 2.87; 95% CI: 1.58-5.21, *P* < 0.001) and the 5-STS test (HR: 2.58; 95% CI: 1.28-5.18, *P* = 0.0057); hypoalbuminemia (HR: 1.5; 95% CI: 0.91-2.48, *P* = 0.11) and the mBMI score (HR: 0.9; 95% CI: 0.58-1.390, *P* = 0.63) had no association with the composite endpoint (Table 2, Supplemental Figures 1 to 3). In the fully adjusted model, 3 of the 6 frailty assessments remained associated with the composite outcome (with Bonferroni adjustment)—these included the CFS (HR: 2.83; 95% CI: 1.61-4.98, *P* < 0.001), FRAIL scale (HR: 1.96; 95% CI: 1.25-3.07, *P* = 0.004), and 5-m gait speed test (HR: 2.8; 95% CI: 1.50-5.25, *P* = 0.001) (Table 2, Central Illustration). For these 3 frailty assessments, a logistic regression analysis displayed relatively linear relationships between higher frailty assessment scores and the log-predicted odds of the composite outcome (Figure 4).

**Table 2:** Association of Frailty With the 1-Year Composite Outcome of All-Cause Mortality and All-Cause Hospitalization

Frailty Assessment	Crude HR	95% CI	*P* Value	Adj HR	95% CI	*P* Value
Clinical Frailty Scale	2.93	1.71-5.02	**<0.001**	2.83	1.61-4.98	**<0.001**
FRAIL scale	2.07	1.33-3.22	**0.001**	1.96	1.25-3.07	**0.004**
5-m gait speed	2.87	1.58-5.21	**<0.001**	2.80	1.50-5.25	**0.001**
5-STS	2.58	1.28-5.18	**0.0057**	2.42	1.20-4.89	0.014
Hypoalbuminemia	1.50	0.91-2.48	0.11	1.36	0.81-2.28	0.24
Modified BMI	0.90	0.58-1.39	0.63	0.99	0.62-1.58	>0.90

Adjusted HR controlled for race and the MAGGIC risk score which incorporates the following variables: age, gender, diabetes mellitus, chronic obstructive pulmonary disease, smoking status, BMI, systolic blood pressure, creatinine, left ventricular ejection fraction, first HF diagnosis within prior 18 mo of baseline visit, NYHA functional class, and current beta-blocker, angiotensin-converting enzyme inhibitor, or angiotensin receptor blocker usage. The **bolded***P* values denotes statistical significance.

Adj = adjusted; other abbreviations as in Table 1.

## Discussion

There are several key insights from this study of 203 community-dwelling patients with HFpEF. First, the prevalence of frailty ranges widely depending on which frailty assessment is used. Second, frailty is increasingly prevalent with advancing HFpEF severity. Third, among the six frailty scales assessed, the CFS, FRAIL scale, and 5-m gait speed tests demonstrated associations with adverse outcomes, whereas the 5-STS, hypoalbuminemia, and the mBMI did not. These findings highlight important differences in existing frailty tools and underscore the importance of additional research to identify optimal frailty tools for evaluating patients with HFpEF.

In our cohort, we found that the prevalence of frailty ranged from 21% to 78% depending on the instrument used. This finding is consistent with prevalence studies of frailty in other populations^12^^,^^34^, ^35^, ^36^, ^37^, ^38^, ^39^ and reflects the scientific landscape of frailty. Despite accepting frailty as an important distinct physiologic condition, there is substantial variation in inter-tool agreement^40^ and limited consensus on defining and characterizing frailty in real-world populations. In general, different tools measure different phenomena and reflect the different definitions of frailty that currently exist. There are two foundational approaches to characterizing frailty: the Fried physical frailty phenotype^41^ and the Rockwood cumulative deficit definition.^42^^,^^43^ The Fried physical frailty phenotype assesses frailty through physical characteristics and abilities based on 5 criteria: unintentional weight loss, self-reported exhaustion, slow walking speed, weakness, and low physical activity. Physical frailty is present when 3 of these 5 criteria are met. In our study, the FRAIL scale, 5-m gait speed test, 5-STS test, hypoalbuminemia, and the mBMI are reflections of the Fried physical frailty phenotype. Meanwhile, the Rockwood cumulative deficit definition of frailty characterizes frailty through a heterogenous combination of decreased cognitive function, mobility, weakness, muscle mass, and poor nutritional status.^44^ In our study, the CFS reflected this definition of frailty. Since there is no consensus agreement on the optimal tool for clinical practice, clinicians do not always use formal or validated criteria when assessing frailty, relying more often on potentially biased subjective processes like the “eyeball test”^45^^,^^46^ which has poor inter-rater reliability.^47^ Our findings here indicate that tools that can formally assess frailty, regardless of whether they measure the Fried physical frailty phenotype or the Rockwood cumulative deficit definition, add value to clinical practice.

Characterizing frailty in HFpEF is particularly challenging given the pathophysiological overlap between these conditions.^2^ In particular, pathogenesis of both conditions is a pro-inflammatory state that triggers mitochondrial dysfunction and oxidative stress.^3^^,^^12^ Additionally, frailty is associated with sarcopenia and circulating inflammatory cytokines, which are features shared with HFpEF.^3^ Consistent with this notion, it is not surprising that the prevalence of frailty increased with increasing severity of HFpEF (defined as either NYHA functional class or MAGGIC score) in our study, which is consistent with prior work among an advanced HFrEF population.^48^ Whether this relationship is due to the biological effects of HF (whereby HF symptoms confound frailty testing) or due to a measurement issue (whereby frailty testing is confounded by HF symptoms) is not known. Indeed, many of the symptoms of frailty (especially those in the physical function domain) are shared with HF. Consequently, when a frailty tool is positive, it may not be clear whether this is because of frailty, HF symptoms, or both. Relatedly, it is frequently unknown whether the detected “frailty” is reversible, such as through the treatment of HF. As the number of therapeutic options expands for HFpEF, understanding whether frailty is present independent of HF symptoms will likely become increasingly important in clinical decision-making. If the impairment is largely driven by HF symptoms, optimizing HF treatment (even via an invasive procedure) may be reasonable; on the other hand, if the impairment supersedes HF symptoms, treating HF may not suffice, and attempting to do so via invasive approaches may carry greater risk than the potential for benefit. Additionally, emerging data suggest that frailty may be a modifiable condition through nonpharmaceutical interventions such as exercise and/or cardiac rehabilitation, further emphasizing the importance of this distinction. The REHAB-HF (Rehabilitation Therapy in Older Acute Heart Failure Patients) trial, and subsequent subgroup analyses of this trial, demonstrate that physical rehabilitation improves physical function and frailty, while also lowering rates of rehospitalization and all-cause mortality, and improving quality of life,^35^^,^^37^ with HFpEF patients displaying the greatest benefit.^36^ The ongoing REHAB-HFpEF trial offers great potential to provide additional insights on the impact of exercise on HFpEF and to better understand the role that frailty plays in this benefit.^49^

We found that the CFS, FRAIL scale, and the 5-m gait speed test were independently associated with the composite endpoint even after adjusting for HF severity. This suggests that these specific tools provide incremental information that goes beyond HF symptoms and severity; whereas factors like hypoalbuminemia and mBMI do not. Moreover, it indicates that these specific tools detect features beyond HF that may reflect the phenomenon of frailty. We found that the association between frailty scores (for the CFS, FRAIL scale, and the 5-m gait speed test) and the composite endpoint was largely linear which makes them easy to interpret—worse scores track with worse outcomes. Additionally, our findings support those reported by Sze et al that found the CFS and 5-m gait speed tests, among nine unique frailty assessments, to have the greatest prognostic value in a HFrEF predominant HF cohort.^14^ In light of their lack of association with outcomes in this study and the fact that albumin levels are influenced by multiple factors (including select comorbid conditions, inflammation, volume status, and diet/nutritional status among others), hypoalbuminemia and mBMI may not be ideal markers for frailty in HFpEF. Instead, given their incremental value for prognosis and ease of administration and interpretation, our findings support the CFS, FRAIL scale, and/or the 5-m gait speed test as reasonable tools to detect frailty in older adults with HFpEF. Importantly, the CFS, FRAIL scale, and 5-m gait speed can each be performed in a matter of seconds with minimal training, further supporting their potential use in routine clinical practice. Findings of frailty could subsequently be used to guide clinical decision-making such as prescribing targeted therapy such as exercise and/or physical rehabilitation interventions currently under investigation.^35^^,^^37^

### Study Limitations

There were several limitations in our study. First, our results are from a single-center specialty program at a quaternary academic center which may have limited generalizability. Although the population of this study is similar to the HFpEF population at large,^50^ additional work in a broader population is needed to validate our findings. Second, our study was limited by a small sample size (n = 203). For example, it is possible that a larger sample size would have revealed associations between other frailty tools and the composite outcome. Larger, geographically diverse studies are needed to validate these findings. Third, our findings are vulnerable to the inherent limitations of chart review. This includes the possibility of inaccurate or missing data related to outcomes of interest including interim hospitalizations and even death. Of note, our institution has a robust electronic medical record system that links to multiple hospitals within the local region and across the country, which mitigates this concern—we excluded 17 patients who lacked any follow-up data. Fourth, we only examined frailty at baseline—future studies may benefit from examining changes in frailty over time as a marker of prognosis.^51^

## Conclusions

Our study demonstrates that different physical frailty assessments yield variations in frailty prevalence estimates and that frailty becomes increasingly prevalent with advancing HFpEF severity. Frailty assessments vary in their prognostic value as the CFS, FRAIL scale, and 5-m gait speed tests are associated with adverse outcomes, while the 5-STS, hypoalbuminemia, and the mBMI scales are not. These results indicate that the CFS, FRAIL scale, or 5-m gait speed tests may be appropriate tools for routine frailty screening in HFpEF, though larger studies are needed to generate consensus agreement on the optimal frailty scale for this population.

Perspectives **COMPETENCY IN MEDICAL KNOWLEDGE:** Physical frailty assessments yield variations in frailty prevalence estimates and prognostic impact. Frailty becomes increasingly prevalent with advancing HFpEF severity. The CFS, the FRAIL scale, and the 5-m gait speed test are associated with adverse outcomes, are quick to administer and easy to interpret, and thus may be reasonable tools for routine frailty assessments in adults with HFpEF.

**TRANSLATIONAL OUTLOOK:** There is no debate regarding the prognostic implications of frailty in HFpEF. Consensus agreement is needed on how to optimally assess frailty in this patient population. Additionally, there is a pressing need for future research to determine if frailty is a modifiable condition that can improve outcomes in patients with HFpEF.

## Funding support and author disclosures

Dr Goyal is supported by 10.13039/100000049National Institute on Aging grant K76AG064428. Dr Damluji has received research funding from the Pepper Scholars Program of the Johns Hopkins University Claude D. Pepper Older Americans Independence Center funded by the 10.13039/100000049National Institute on Aging P30-AG021334; mentored patient-oriented research career development award from the 10.13039/100000050National Heart, Lung, and Blood Institute K23-HL153771; The NIH National Institute of Aging R01-AG078153; and the 10.13039/100006093Patient-Centered Outcomes Research Institute (PCORI). Dr Pastva is supported through the R01AG078153 and P30AG028716 grants. All other authors have reported that they have no relationships relevant to the contents of this paper to disclose.
