# Supplementary material for: Comparison of Physical Frailty Assessments in Heart Failure With Preserved Ejection Fraction
Source: JACC Adv. 2024 Dec 26;3(12):101395. doi: 10.1016/j.jacadv.2024.101395 (PMC11683403; doi:10.1016/j.jacadv.2024.101395)

**Supplemental Figure 1:** Kaplan-Meier curve for the composite outcome of all-cause mortality and all-cause hospitalization in patients with (5-STS ≥ 15 seconds) and without (5-STS < 15 seconds) frailty.

5-STS: 5 timed sit-to-stand test


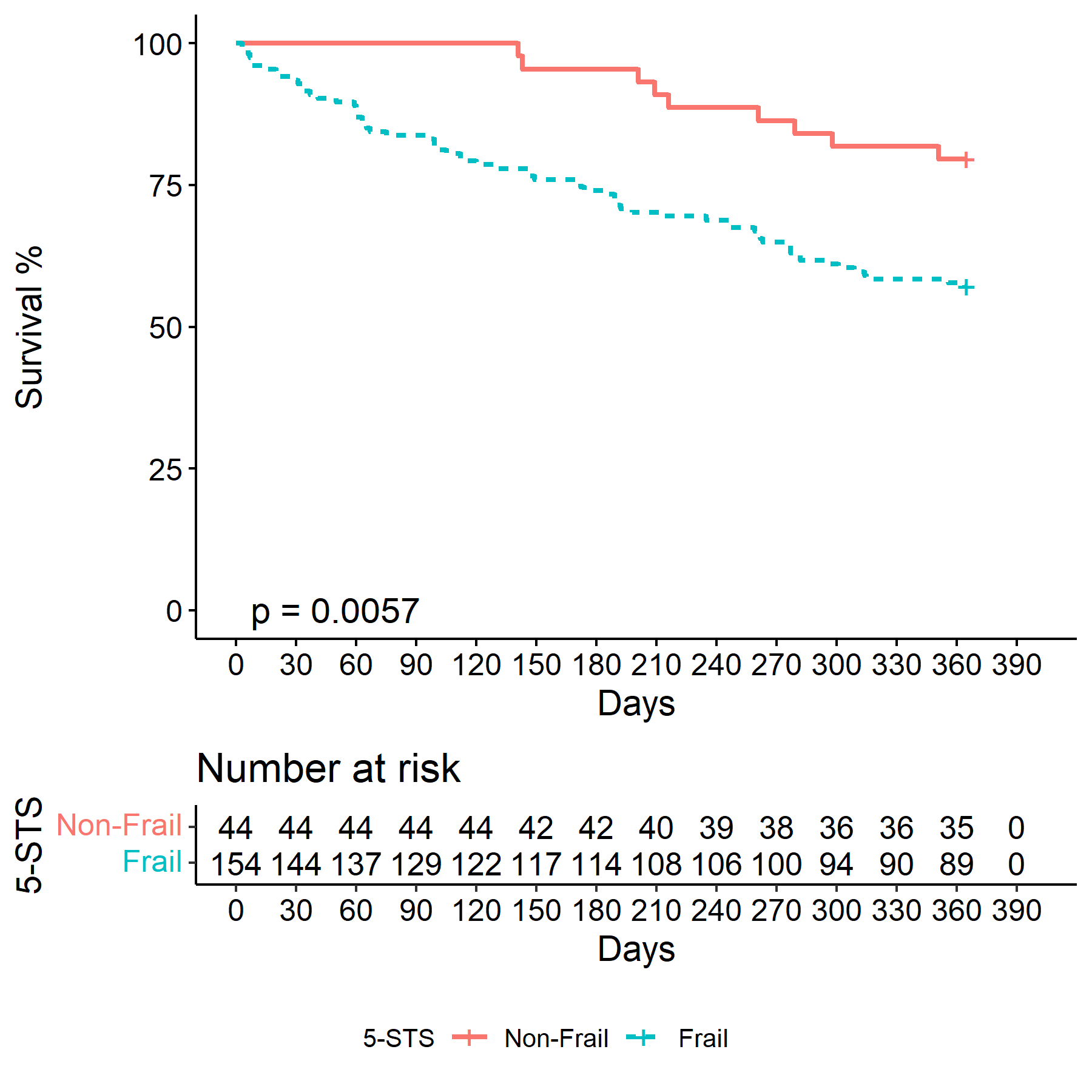


**Supplemental Figure 2:** Kaplan-Meier curve for the composite outcome of all-cause mortality and all-cause hospitalization in patients with (albumin < 3.5 g/dL) and without (albumin ≥ 3.5 g/dL) frailty.


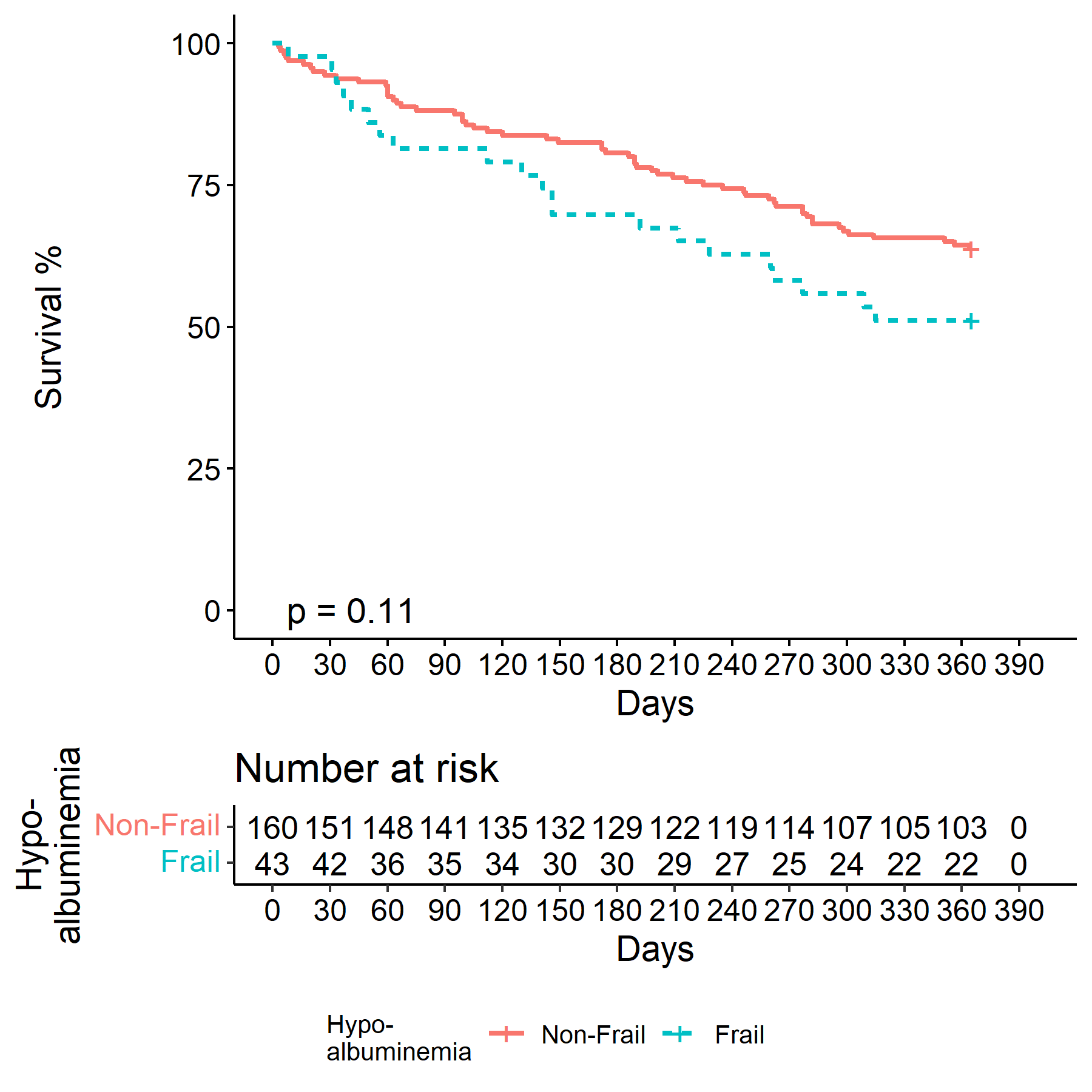


S

**Supplemental Figure 3:** Kaplan-Meier curve for the composite outcome of all-cause mortality and all-cause hospitalization in patients with (mBMI < 119.0 ) and without (mBMI ≥ 119.0) frailty.

mBMI: Modified body mass index


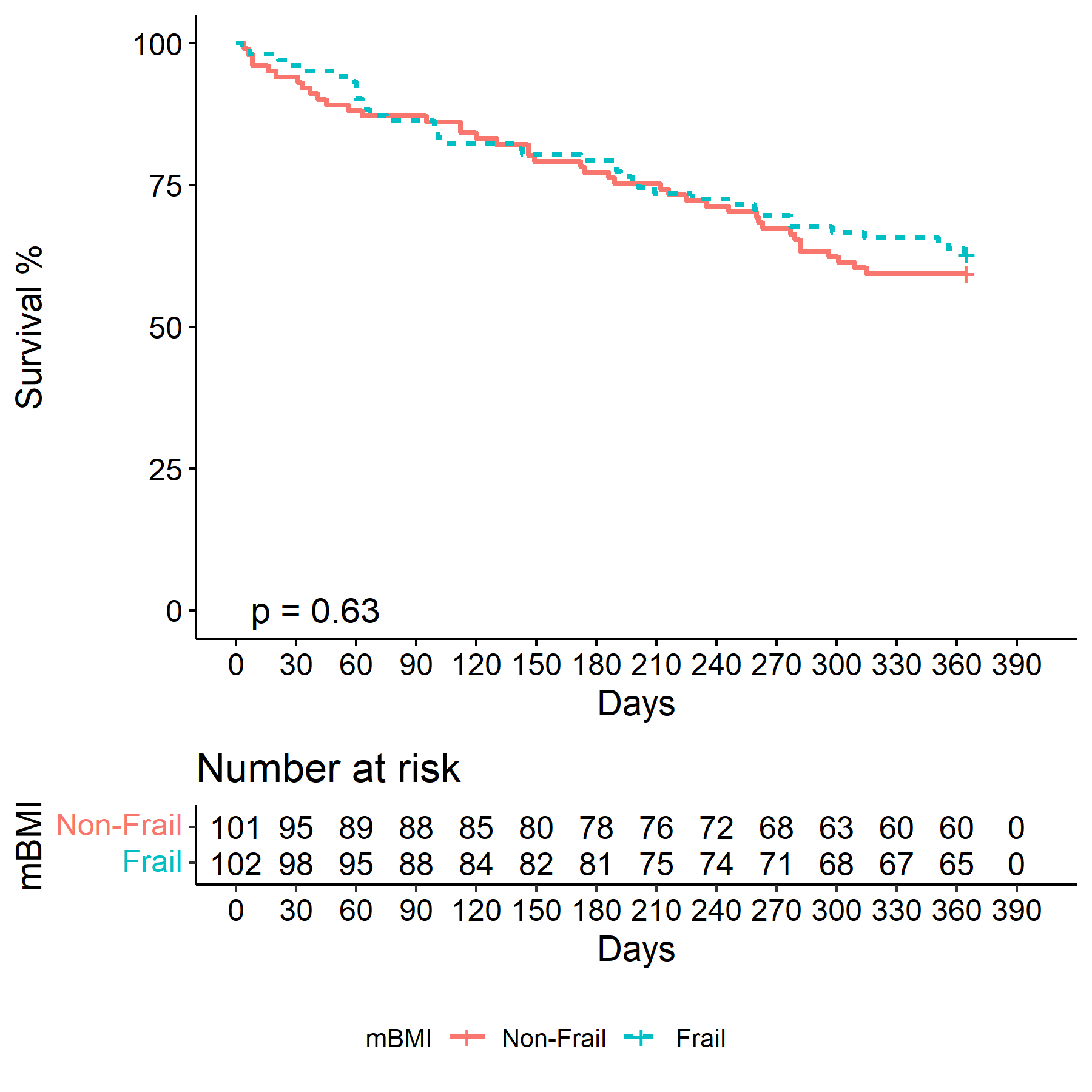

Supplement: Supplemental material [file mmc1.docx]
